# Serum metabolomics analysis for the progression of esophageal squamous cell carcinoma

**DOI:** 10.7150/jca.54429

**Published:** 2021-04-02

**Authors:** Xia Li, Lihong Zhao, Mengke Wei, Jiali Lv, Yawen Sun, Xiaotao Shen, Deli Zhao, Fuzhong Xue, Tao Zhang, Jialin Wang

**Affiliations:** 1 Department of Biostatistics, School of Public Health, Cheeloo College of Medicine, Shandong University, Jinan 250012, China.; 2 Tumor Preventative and Therapeutic Base of Shandong Province, Feicheng People's Hospital, Feicheng 271600, China.; 3 Shandong Cancer Hospital and Institute, Shandong First Medical University and Shandong Academy of Medical Sciences, Jinan 250117, China.; 4 Interdisciplinary Research Center on Biology and Chemistry, and Shanghai Institute of Organic Chemistry, Chinese Academy of Sciences, Shanghai 200032, China.

**Keywords:** esophageal squamous cell carcinoma, serum metabolites, progression, FCM, ordinal logistic regression.

## Abstract

**BACKGROUND:** Previous metabolomics studies have found differences in metabolic characteristics between the healthy and ESCC patients. However, few of these studies concerned the whole process of the progression of ESCC. This study aims to explore serum metabolites associated with the progression of ESCC.

**METHODS:** Serum samples from 653 participants (305 normal, 77 esophagitis, 228 LGD, and 43 HGD/ESCC) were examined by ultra-high performance liquid chromatography quadruple time-of-flight mass spectrometry (UHPLC-QTOF/MS). Principal component analysis (PCA) was first applied to obtain an overview of the clustering trend for the multidimensional data. Fuzzy c-means (FCM) clustering was then used to screen metabolites with a changing tendency in the progression of ESCC. Univariate ordinal logistic regression analysis and multiple ordinal logistic regression analysis were applied to evaluate the association of metabolites with the risk of ESCC progression, and adjusted for age, gender, BMI, tobacco smoking, and alcohol drinking status.

**RESULTS:** After FCM clustering analysis, a total of 38 metabolites exhibiting changing tendency among normal, esophagitis, LGD, and HGD/ESCC patients. Final results showed 15 metabolites associated with the progression of ESCC. Ten metabolites (dopamine, L-histidine, 5-hydroxyindoleacetate, L-tryptophan, 2'-O-methylcytidine, PC (14:0/0:0), PC (O-16:1/0:0), PE (18:0/0:0), PC (16:1/0:0), PC (18:2/0:0)) were associated with decreased risk of developing ESCC. Five metabolites (hypoxanthine, inosine, carnitine (14:1), glycochenodeoxycholate, PC (P-18:0/18:3)) were associated with increased risk of developing ESCC.

**CONCLUSIONS:** These results demonstrated that serum metabolites are associated with the progression of ESCC. These metabolites are capable of potential biomarkers for the risk prediction and early detection of ESCC.

## Introduction

Esophageal squamous cell carcinoma (ESCC) is the predominant subtype of esophageal carcinoma, accounts for about 90% of cases of esophageal carcinoma worldwide 1, 2. Most global ESCC cases occurred in the Central and South-East Asian region, which called “Asian esophageal cancer belt”, such as Iran, Turkey, Kazakhstan, and China 3, 4. ESCC has a long progressive stage, including esophagitis, basal cell hyperplasia (BCH), esophageal squamous dysplasia (ESD), cancer *in situ*, invasive cancer, and metastatic cancer 5. Unfortunately, it is asymptomatic in the early stage, the majority of patients are diagnosed at an advanced stage, with a poor prognosis. So identifying patients before they progress to advanced-stage disease is very important. Currently, early detection and screening of ESCC is primarily based on endoscopy, biopsy and pathological examination 6. Endoscopic examination with iodine staining is widely used in high-risk areas of China 7, 8. However, this method is expensive and invasive; it is difficult to be accepted by general residents 9, 10. New cost-effective and non-invasive methods for identifying these patients with high sensitivity and specificity are not available.

Metabolic reprogramming has been proposed to be a key hallmark of cancer 11. Recently, the application of metabolomics towards cancer research has been paid more attention. Metabolomics is a comprehensive and quantitative analysis of metabolites in the biological system under study 12. Currently, metabolomics has been a powerful tool in the identification of metabolic changes in cancer progression and the discovery of non-invasive biomarkers for cancer prediction and diagnosis 13-16. However, previous metabolomics studies on ESCC mainly focused on exploring potential diagnostic biomarkers based on healthy controls and ESCC patients^17^-^21^, few studies pay attention to the whole process of the progression of ESCC. Zhang et al.^22^ used serum metabolomic strategy based on gas chromatography-mass spectrometry (GC-MS) to identify and validate potential metabolic markers for the discrimination of normal control, ESCC and ESD patients, it showed reasonable performance. Nevertheless, they still chose the traditional two-group comparison method in screening differential metabolites and ignored the ordinal relationship about disease progression.

In this study, serum samples from 653 participants (305 normal, 77 esophagitis, 228 LGD, and 43 HGD/ESCC) were collected at the Esophageal Cancer Screening Base in the high-risk area of China, covering the whole progression of ESCC. An untargeted ultra-high performance liquid chromatography quadruple time-of-flight mass spectrometry (UHPLC-QTOF/MS) metabolomics approach was applied to these serum samples. Fuzzy c-means (FCM) clustering analysis and ordinal logistic regression analysis were combined to identify serum metabolites associated with the progression of ESCC. This study could be helpful for discovering new biomarkers for risk prediction and early detection of ESCC, especially precancerous lesions.

## Materials and Methods

### Study population

This study included subjects aged 40 to 69 years who had screened for esophageal cancer at the Esophageal Cancer Screening Base of Shandong Province (City of Feicheng, Shandong, China) between June 2013 and September 2014. The study was approved by the Ethics Committee of the Shandong Cancer Hospital and Institute, and all participants signed written informed consent.

The inclusion criteria for all patients in this study were as follows: (1) Diagnosis of esophageal squamous epithelial lesions at the Esophageal Cancer Screening Base of Shandong Province between June 2013 and September 2014. (2) Aged 40-69 years.

The exclusion criteria were as follows: (1) Coexisting other malignant tumors. (2) Having history of surgery, radiotherapy or chemotherapy for other malignant tumors. (3) Suffering from metabolic diseases, liver diseases or kidney diseases. (4) Taking take any medications. (5) Patients with inadequate clinical information.

The questionnaire interview was conducted by trained investigators to obtain their information, including age, gender, body mass index (BMI), tobacco smoking, and alcohol drinking status. In this study, all participants underwent a simple physical examination and endoscopy with mucosal iodine staining. For the participants who had suspicious tissues in esophageal mucosa (iodine-negative), the non-staining tissues were taken for biopsy and underwent pathological evaluation by two pathologists.

ESD is subdivided into three levels of severity: mild, moderate, and severe. Mild and moderate ESD are combined to low-grade dysplasia (LGD), while severe ESD and cancer *in situ* are considered as high-grade dysplasia (HGD). In this study, a total of 305 healthy subjects, 77 patients with esophagitis, 188 patients with mild ESD, 40 patients with moderate ESD, 15 patients with severe ESD, 12 patients with cancer *in situ*, and 16 patients with ESCC were enrolled. We grouped the participants into 4 groups: 'normal', 'esophagitis', 'LGD', and 'HGD/ESCC'. For the participants with multiple diagnoses in one biopsy, we only care about the most severe one.

### Serum samples collection and UHPLC-QTOF/MS analysis

In brief, blood samples were taken in the morning from the participants after an overnight fasting. Whole blood specimens were immediately processed to obtain the serum samples and were immediately stored at -80°C until further analysis. Before UHPLC-QTOF/MS analysis, serum samples were thawed at 4 °C on ice and underwent the preprocessing procedure. The serum samples were randomly injected for the UHPLC-QTOF/MS analysis. A more detailed description can be found in the previous paper 17. Blank samples (75 % ACN in water) and QC samples were injected every eight samples during acquisition. Then raw data obtained by UHPLC-QTOF/MS analysis was further preprocessed and annotated (see details in supplement materials). Finally, 341 metabolite features were obtained for statistical analysis.

### Statistical analysis

Principal component analysis (PCA) was first applied to obtain an overview of the clustering trend for the multidimensional data and evaluate the overall stability of the metabolomics data. Fuzzy c-means (FCM) clustering was then used to screen metabolites with a changing tendency in the progression of ESCC.

FCM 23, 24 is a kind of data soft clustering techniques, it allows each data point to belong to multiple clusters. Each data point has a fuzzy degree of belonging to each cluster. The algorithm of FCM is based on minimize an objective function:


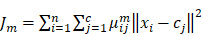
, 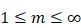
(1)

Where, *μ_ij_* is the degree to which an observation *x_i_* belongs to a cluster *c_j_*, *c_j_* is the center of the cluster *j*, *m* is a hyper-parameter that controls the level of fuzzy cluster fuzziness. The whole algorithm is made up of three steps: (a) Specify a number of clusters. (b) Assign coefficients randomly to each data point for being in the clusters. (c) Repeat until the algorithm has converged: Compute the centroid for each cluster, using the formula (2). For each data point, compute its coefficients of being in the clusters, using the formula (3).


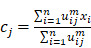
(2)


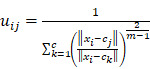
(3)

Then the univariate ordinal logistic regression analysis was performed to determine the significance of each metabolite, and the relevant false discovery rates (FDR) based on the p values were estimated in the context of multiple testing. Metabolites with FDR value less than 0.05 were further adjusted for age, gender, BMI, tobacco smoking, and alcohol drinking status. Finally, all metabolites founded above and five covariates mentioned above were included in multiple ordinal logistic regression analysis. All of the statistical analyses were performed on the R platform (version 3.5.3).

## Results

### General Characteristics of Study Participants

Table 1 shows the baseline characteristics of study participants included in this study. The mean ages calculated for the normal, esophagitis, LGD, and HGD/ESCC groups were 52.91, 58.31, 58.24, and 62.47 years, respectively. The univariate ordinal logistic analysis showed that significant differences (*P* < 0.05) in age, BMI, tobacco smoking, and alcohol drinking status among these groups. Notably, the highest percentages for tobacco smoking and alcohol drinking status were recorded in the HGD/ESCC group.

### Metabolic Profiling of Serum Samples

In the UHPLC-QTOF-MS dataset, a total of 8182 metabolite features were detected. First, the reproducibility of the polar metabolite features was evaluated by relative standard deviation (RSD); metabolite features showing large variations were removed (RSD > 30%) before conducting the statistical analysis. After annotation, we obtained 341 metabolite features. To determine whether the metabolic profiles of the four groups were different, a multivariate statistical analysis using the principal component analysis (PCA) model was conducted. The score plots obtained from the PCA models are presented in Fig. S1. We excluded 12 PCA-based-outlier serum samples (7 normal, 2 esophagitis, 3 LGD) from further analysis. The PCA scores plot shows a clear cluster of the QC sample, indicating the high stability and reproducibility of the instrument.

### FCM clustering analysis of metabolites

**Figure 1:**
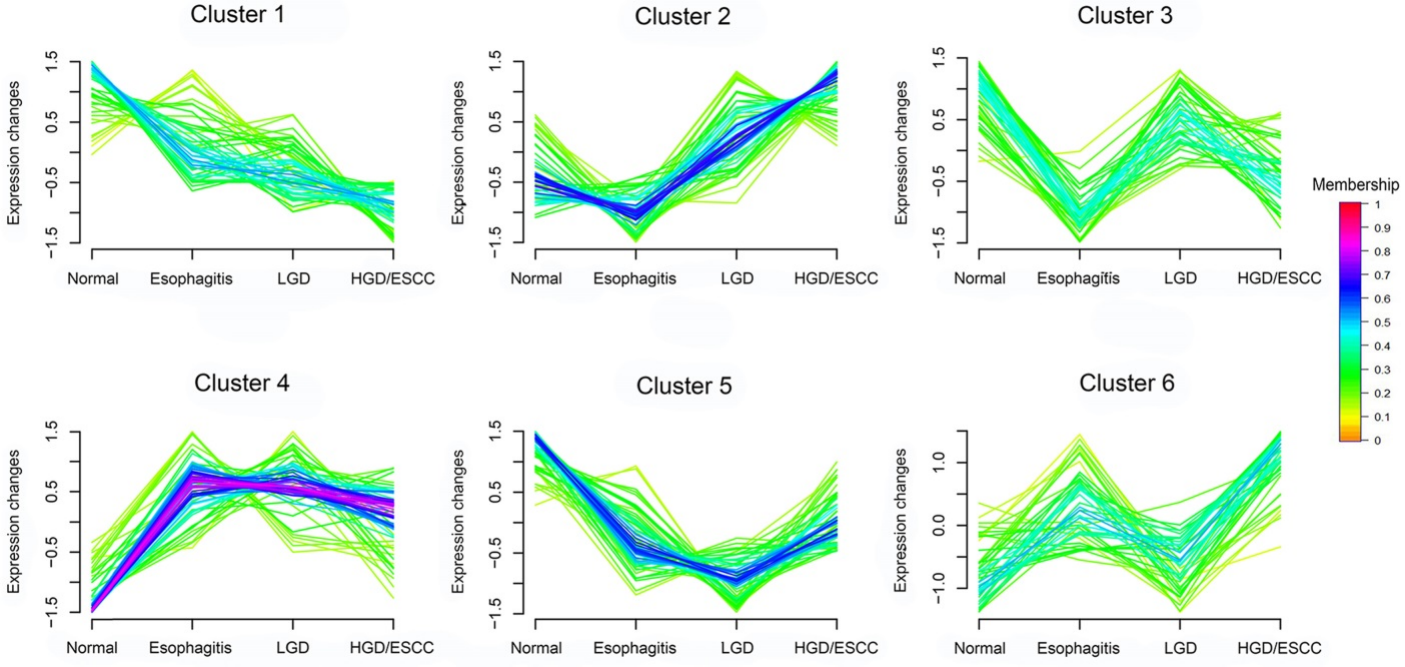
FCM clustering analysis of 341 metabolites from normal participants to HGD/ESCC patients. LGD, low-grade dysplasia; HGD, high-grade dysplasia; ESCC, esophageal squamous cell carcinoma. Each of these lines represents a metabolite. The color of the line indicates the membership of the metabolite in cluster.

According to changing tendency, metabolites in the progression of ESCC were divided into six categories by FCM clustering analysis (Fig. 1). First, we excluded metabolites in cluster3 and cluster6 because they don't change regularly. Then, metabolites in cluster4 and cluster5 did not change significantly in the esophagitis, LGD, and HGD/ESCC groups, they were also excluded. Finally, 38 metabolites with membership greater than 0.45 in cluster 1 and cluster 2 were selected for further analysis.

### Metabolites associated with the risk of ESCC Progression

**Figure 2:**
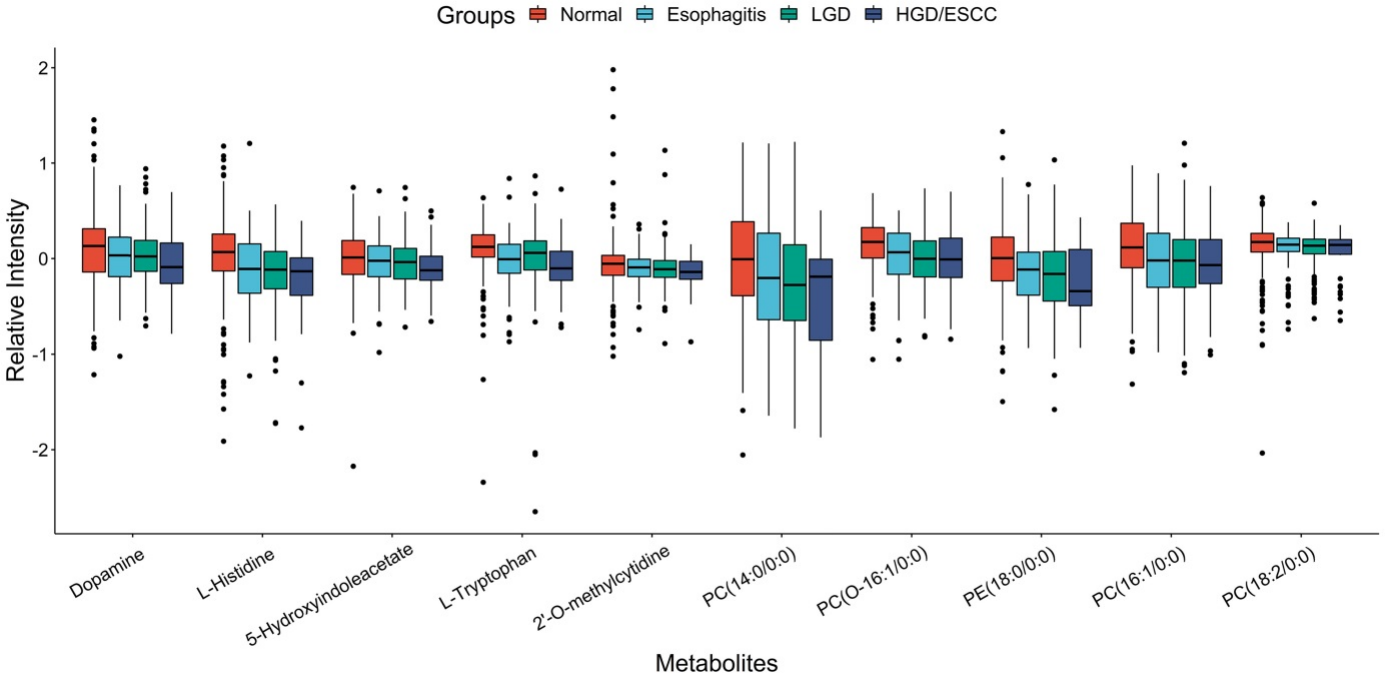
Boxplots of relative intensity of expression of ten metabolites that associated with decreased risk of developing ESCC between normal and esophagitis, LGD and HGD/ESCC patients. LGD, low-grade dysplasia; HGD, high-grade dysplasia; ESCC, esophageal squamous cell carcinoma.

After univariate ordinal logistic regression analysis, 15 metabolites were significant (FDR < 0.05) among with 38 metabolites mentioned above (Table S1). Then, adjusted age, gender, BMI, tobacco smoking, and alcohol drinking status, their expression were remains associated with the risk of ESCC progression (Table 2). Ten metabolites (dopamine, L-histidine, 5-hydroxyindoleacetate, L-tryptophan, 2'-O-methylcytidine, PC (14:0/0:0), PC (O-16:1/0:0), PE (18:0/0:0), PC (16:1/0:0), PC (18:2/0:0)) were associated with decreased risk of developing ESCC. Their boxplots of relative intensity of expression in the esophagitis, LGD, and HGD/ESCC groups were depicted in Fig. 2. Five metabolites (hypoxanthine, inosine, carnitine (14:1), glycochenodeoxycholate, PC (P-18:0/18:3)) were associated with increased risk of developing ESCC. Their changes of relative intensity of expression in each group are shown in Fig. S2.

**Figure 3:**
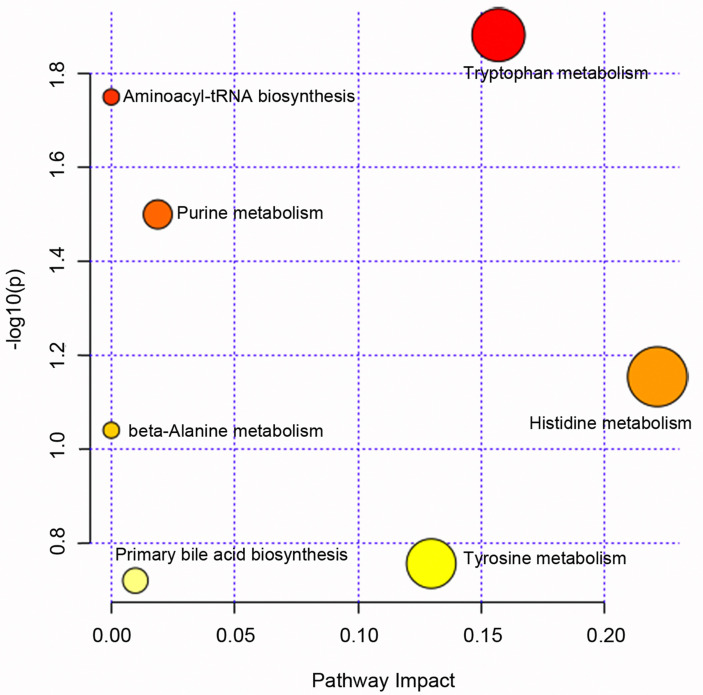
Pathway enrichment analysis for metabolites that associated with the progression of esophageal squamous cell carcinoma.

Pathway enrichment analysis for metabolites that associated with the progression of ESCC was performed; we can see these metabolites main involved tryptophan metabolism, purine metabolism, histidine metabolism et al pathway (Fig. 3, see details in Table S2).

Further multiple ordinal logistic regression analysis included all 15 metabolites founded by above analysis and age, gender, BMI, tobacco smoking, and alcohol drinking status. After adjustment, several metabolites (dopamine, L-Histidine, 5-Hydroxyindoleacetate, PC (O-16:1/0:0), hypoxanthine, Inosine, glycochenodeoxycholate) were remain associated with the progress of ESCC (Table 3).

## Discussion

In the process of normal esophageal squamous epithelium develops into cancer, body metabolism also changes. In this study, we used untargeted metabolomics study based on UHPLC-QTOF/MS technique to explore serum metabolites associated with the progression of ESCC. A total of 15 metabolites associated with the progression of ESCC. These metabolites related to purine metabolism and energy metabolism (fatty acid metabolism and amino acid metabolism).

A series of phospholipid metabolites were found dysregulated in lesions associated with ESCC, mainly phosphatidylcholines (PC). Phospholipids converted from choline, they can form the bilayer structures of all cell membranes and are an important part of all cells 25. Ma et al. 26 reported the serum expression levels of PCs in ESCC patients were lower than healthy subjects. In another serum metabolomics study, PCs were a major class of dysregulated metabolites, both down-regulated and up-regulated, suggesting potential perturbation of phosphocholine metabolism in ESCC 27. Reduced PC metabolism has been also observed in prostate cancer 28 and cervical cancer 29. Most of the PCs found in this study down-regulated with the development of ESCC. This is consistent with previous studies. The reduced metabolism of PCs in patients may meet the needs of high proliferation of cancer cells.

Carnitine is an important metabolite in fatty acid β-oxidation, it transports acyl CoA across the mitochondrial membranes into the mitochondria for oxidative decomposition 30. The present result indicated that fatty acid β-oxidation was disturbed in the process of developing ESCC. Xu et al. 31 also found that L- carnitine up-regulated in ESCC patients compared to healthy controls. Fatty acid β-oxidation provides extra ATP and is an important source of NADPH in cancer cells metabolic reprogramming 32. The increased carnitine promotes fatty acid β-oxidation, which helps the development of cancer.

We observed that metabolites from the purine metabolic pathway, hypoxanthine and inosine were associated with ESCC risk. In the purine degradation pathway, inosine can convert to hypoxanthine by purine nucleoside phosphorylase (PNP), and then xanthine-oxidase (XO) catalyzes hypoxanthine oxidation to form uric acid finally 33. Previous studies found an up-regulated uric acid in ESCC patients' urine samples 18 and plasma samples 31. This change in expression also showed in serum samples of esophageal cancer patients, gastric cancer patients and colorectal cancer patients 34. Previous studies also showed that uric acid contributes to cancer risk, recurrence, and mortality 35.

Glycochenodeoxycholate is a primary bile acid; it showed an up-regulated trend in the process of developing ESCC in the present study. Nishioka et al.^36^ reported the possibility of bile acid contributing to squamous carcinogenesis of the esophagus, it promotes the proliferation of ESCC.

In the present study, L-tryptophan and 5-hydroxyindoleacetate were down-regulated expression in lesions associated with ESCC. Zhu et al. 20 also reported the plasma levels of L-tryptophan were lower in ESCC patients. 5-hydroxyindoleacetate is a product of L-tryptophan metabolism in tryptophan metabolic pathway. Compared to the healthy controls, the concentration of tryptophan is down-regulated in both ESCC and metastatic ESCC patients, further analysis showed that disturbed tryptophan metabolism correlating to progression and metastasis of ESCC 37, 38. The level of tryptophan decreased in the esophageal cancer (EC) and esophageal adenocarcinoma (EAC) patients compared with healthy controls, which indicates an increased demand for and overutilization of amino acids in the tumor tissue 39, 40.

Ma et al. 41 have reported that the expression of histidine is significantly down-regulated in ESCC patients compared to healthy people. Wang et al. 42 also found that histidine significantly decreased in EC tissues in comparison to normal mucosae. In this study, L-histidine also has a down-regulated trend in the development of ESCC. Histidine catabolism produces one carbon unit to participate in the synthesis of purines and pyrimidines. The lower levels of histidine in body mainly due to active nucleic acid metabolism in tumor cells, histidine consumption are markedly increased.

Dopamine is involved in the tyrosine metabolic pathway. We found it down-regulated in the development of ESCC, possibly because of a decrease in tyrosine. Tyrosine down-regulated has also been observed in EC and EAC patients 39, 40.

Although our study included 653 serum samples from the normal to ESCC progression stages (305 normal, 77 esophagitis, 228 LGD, and 43 HGD/ESCC) and used ordinal information of these four groups in statistical analysis to explore serum metabolites associated with the progression of ESCC. This study also has some limitations. First, the distribution of sample size in each group was unbalanced, which could affect the results of our statistical analysis. Second, this study found some metabolites related to the progress of ESCC, but because it is difficult to obtain more samples covering all stages of ESCC, the role of these metabolites in risk prediction and early detection of ESCC lacks further analysis and validation.

In conclusion, this serum metabolomics study demonstrated that serum metabolites associated with the progression of ESCC. The results obtained in the current study showed that 15 metabolites were significantly altered among normal, esophagitis, LGD and HGD/ESCC groups. These metabolites are capable of potential biomarkers for the risk prediction and early detection of ESCC. Further studies are necessary in order to fully understand the association of metabolic changes with ESCC progression and their risk prediction and early detection potentials.

## Supplementary Material

Supplementary figures and tables.

## Figures and Tables

**Table 1:** General characteristics of study subjects

Variables	Normal	Esophagitis	LGD	HGD/ESCC	*P*-value
N	305	77	228	43	
Age (years) (SD)	52.9 (7.6)	58.3 (6.9)	58.2 (7.3)	62.5 (6.4)	<0.001
Female, n (%)	175 (57.4)	39 (50.7)	114 (50.0)	21 (48.8)	0.068
BMI (Kg/m^2^) (SD)	24.8 (3.4)	23.6 (3.7)	24.2 (3.2)	23.1 (3.6)	0.002
Tobacco smoking, n (%)	53 (17.4)	18 (23.4)	61 (26.8)	14 (32.6)	0.002
Alcohol drinking, n (%)	75 (24.6)	23 (29.9)	69 (30.2)	18 (41.9)	0.024

Abbreviations: BMI, body mass index; SD, standard deviation; LGD, low-grade dysplasia; HGD, high-grade dysplasia; ESCC, esophageal squamous cell carcinoma.

**Table 2:** Metabolites associated with the progression of ESCC by ordinal logistic analysis adjusted for age, gender, BMI, tobacco smoking and alcohol drinking status.

Metabolites	Cluster	Adjusted OR (95% CI)	p value
Dopamine	1	0.70 ( 0.59 ~ 0.84 )	1.67E-04
L-Histidine	1	0.60 ( 0.50 ~ 0.72 )	9.03E-08
5-Hydroxyindoleacetate	1	0.72 ( 0.61 ~ 0.84 )	5.08E-05
L-Tryptophan	1	0.73 ( 0.62 ~ 0.85 )	8.65E-05
2'-O-methylcytidine	1	0.72 ( 0.53 ~ 0.97 )	3.02E-02
PC (14:0/0:0)	1	0.68 ( 0.58 ~ 0.81 )	9.13E-06
PC (O-16:1/0:0)	1	0.59 ( 0.50 ~ 0.69 )	2.06E-10
PE (18:0/0:0)	1	0.59 ( 0.49 ~ 0.71 )	2.26E-08
PC (16:1/0:0)	1	0.64 ( 0.54 ~ 0.76 )	2.64E-07
PC (18:2/0:0)	1	0.74 ( 0.63 ~ 0.87 )	2.32E-04
Hypoxanthine	2	1.37 ( 1.17 ~ 1.59 )	7.21E-05
Inosine	2	1.35 ( 1.16 ~ 1.56 )	9.11E-05
Carnitine (14:1)	2	1.23 ( 1.05 ~ 1.43 )	9.52E-03
Glycochenodeoxycholate	2	1.22 ( 1.05 ~ 1.42 )	1.09E-02
PC (P-18:0/18:3)	2	1.20 ( 1.03 ~ 1.41 )	1.85E-02

**Table 3:** Multiple ordinal logistic regression analysis for association between the progression of ESCC and all metabolites.

Metabolites	*β*	Std. Error	t value	p value	OR (95% CI)
Dopamine	-0.213	0.099	-2.157	3.10E-02	0.81 ( 0.67 ~ 0.98 )
L-Histidine	-0.365	0.101	-3.601	3.17E-04	0.69 ( 0.57 ~ 0.85 )
5-Hydroxyindoleacetate	-0.230	0.094	-2.463	1.38E-02	0.79 ( 0.66 ~ 0.95 )
L-Tryptophan	-0.056	0.096	-0.582	5.60E-01	0.95 ( 0.78 ~ 1.14 )
PC(14:0/0:0)	0.179	0.136	1.322	1.86E-01	1.20 ( 0.92 ~ 1.56 )
PC(O-16:1/0:0)	-0.251	0.103	-2.425	1.53E-02	0.78 ( 0.64 ~ 0.95 )
PE(18:0/0:0)	-0.231	0.120	-1.920	5.49E-02	0.79 ( 0.63 ~ 1.00 )
PC(16:1/0:0)	-0.212	0.135	-1.573	1.16E-01	0.81 ( 0.62 ~ 1.05 )
Hypoxanthine	0.366	0.084	4.372	1.23E-05	1.44 ( 1.22 ~ 1.70 )
PC(18:2/0:0)	-0.106	0.101	-1.053	2.92E-01	0.90 ( 0.74 ~ 1.10 )
Inosine	0.348	0.085	4.106	4.02E-05	1.42 ( 1.20 ~ 1.67 )
Carnitine(14:1)	0.065	0.089	0.729	4.66E-01	1.07 ( 0.90 ~ 1.27 )
Glycochenodeoxycholate	0.258	0.085	3.037	2.39E-03	1.29 ( 1.10 ~ 1.53 )
2'-O-methylcytidine	-0.154	0.117	-1.312	1.90E-01	0.86 ( 0.68 ~ 1.08 )
PC(P-18:0/18:3)	0.144	0.081	1.771	7.66E-02	1.16 ( 0.98 ~ 1.36 )

## Abbreviations

ESCC: Esophageal squamous cell carcinoma
LGD: low-grade dysplasia****
HGD: High-grade dysplasia****
BMI: body mass index****
RSD: relative standard deviation
PCA: Principal component analysis
FCM: Fuzzy c-means clustering
FDR: false discovery rates
OR: odds ratio
CI: confidence interval
