# Supplementary material for: Serum metabolomics analysis for the progression of esophageal squamous cell carcinoma
Source: J Cancer. 2021 Apr 2;12(11):3190–7. doi: 10.7150/jca.54429 (PMC8100812; doi:10.7150/jca.54429)
Supplement: Supplementary file 1 — Supplementary figures and tables. [file jcav12p3190s1.pdf]

**Supplementary materials**

**Serum metabolomics analysis for the progression of esophageal squamous cell carcinoma**

**Serum samples collection and preparation**

Blood samples were taken in the morning from the participants after an overnight fasting. The samples were clotted at 37 °C water batch for 30 min and followed by centrifugation for 15 min at 3000 rpm. Then the serum supernatant was taken, immediately stored at -80°C until further analysis.

Before UHPLC-QTOF/MS analysis, serum samples were thawed at 4 °C on ice. Then 50 μL of serum sample was taken and placed in a 96-well plate, then extracted with 150 μL of MeOH (which was kept at -20 °C before extraction) using Bravo liquid handling system (Agilent Technologies, USA), and followed by vortex for 30 s and incubation for 2 h at -20 °C to precipitate proteins. The 96-well plate was then centrifuged at 4000 rpm for 20 min at 4 °C. The resulting supernatants were transferred to LC–MS vials and stored at -80 °C until the UHPLC-QTOF/MS analysis.

**UHPLC-QTOF/MS analysis**

The serum samples were randomly injected for the UHPLC-QTOF/MS analysis. Quality control (QC) samples were prepared by pooling aliquots of all serum samples that were representative of the serum samples under analysis, and used for data normalization. Blank samples (75 % ACN in water) and QC samples were injected every eight samples during acquisition.

The UHPLC-QTOF/MS analyses were performed using a UHPLC system (1290 series, Agilent Technologies, USA) coupled to a quadruple time-of-flight (QTOF) mass spectrometer (Agilent 6550 iFunnel Q-TOF, Agilent Technologies, USA). Waters ACQUITY UHPLC HSS T3 columns [particle size, 1.8 μm; 100 mm (length) × 2.1 mm (i.d.)] were used for the LC separation and the column temperature was kept as 25 °C. The flow rate was 0.5 mL/min and the sample injection volume was 6 μL. The mobile phases A was 0.1 % FA in water in positive mode (ESI+) or 0.5 mM $NH_4F$ in water in negative mode (ESI-), and B was 0.1 % FA in ACN in positive mode or 100 % ACN in negative mode. The linear gradient was set as follows: 0–1 min: 1 % B, 1–8 min: 1 % B to 100 % B, 8–10 min: 100 % B, 10–10.1 min: 100 % B to 1 % B, 10.1–12 min: 1 % B. The acquisition rate was set as 4 spectra/s and the TOF mass range was set as m/z 50–1200 Da. The parameters of MS data acquisition were set as follows: sheath gas temperature, 400 °C; dry gas temperature, 250 °C; sheath gas flow, 12 L/min; dry gas flow, 16 L/min; capillary voltage, 3000 V in positive mode or -3000 V in negative mode, respectively; nozzle voltage, 0 V; and nebulizer pressure, 20 psi in positive or 40 psi in negative mode, respectively.

Tandem mass spectrometry (MS/MS) data acquisition was performed using another quadruple time-of-flight mass spectrometer (Triple TOF 5600+, AB SCIEX, USA). QC samples were used for MS/MS data acquisition. To expand the coverage of MS/MS spectra, the mass range were divided into four segments: 50–300 Da, 290–600 Da, 590–900 Da, 890–1200 Da. The acquired MS/MS spectra were matched against in-house tandem MS spectral library for metabolite identification (see details in follow). The source parameters were set as

follows: GAS1, 60; GAS2: 60; CUR: 30; TEM: 600 °C; ISVF: 5500 V and -4500 V in positive and negative modes, respectively.

**Metabolite identification**: Tandem mass spectrometry (MS/MS) spectra data for metabolites were acquired using AB Sciex TripleTOF 5600+. The acquired MS/MS spectra were matched against our in-house standard MS/MS spectral library and metabolite standards. The MS/MS spectra match score was calculated using dot-product algorithm ranging from 0-1. The cutoff for match score was set as 0.8. The MS/MS spectra match results were further manually checked to confirm the identification. All the m/z errors are less than 25 ppm and all the RT errors are less than 18 seconds. Secondly, the biomarkers whose MS/MS spectra were not matched in our in-house databases or cannot be interpreted by MS/MS spectra, were searched against online databases of HMDB (www.hmdb.ca), METLIN (http://metlin.scripps.edu/) and KEGG (http://www.genome.jp/kegg/). The mass tolerance between the measured m/z values and the exact masses of the components of interest was set to within 30 ppm. The potential biomarkers were identified according to their molecular weights.

**Data preprocessing and annotation**

Raw data obtained by UHPLC-QTOF/MS analysis was firstly converted to the mzXML format using ProteoWizard, and processed by R package XCMS (version 3.2). Then a data matrix consisted of the retention time (RT), mass-to-charge ratio (m/z) values, and peak intensity was generated. R package CAMERA was used for peak annotation after XCMS data processing. Metabolic features detected less than 80 % in all the QC samples were discarded. Only monoisotopic peaks annotated by CAMERA were selected for the subsequent statistical analyses. To remove the unwanted analytical variations occurring intra- and inter-batches, each metabolite peak in all subject samples was normalized using the LOESS method based on QC samples. Then the relative intensity of each metabolite was standardized with Z-transformation (mean = 0, SD = 1).

**Supplementary figure legends:**

**Figure S1**. The PCA performed on the whole samples including normal participants, esophagitis, LGD and HGD/ESCC patients, and QC samples. LGD, low-grade dysplasia; HGD, high-grade dysplasia; ESCC, esophageal squamous cell carcinoma.

**Figure S2**. Boxplots of relative intensity of expression of five metabolites that associated with increased risk of developing ESCC between normal and esophagitis, LGD and HGD/ESCC patients. LGD, low-grade dysplasia; HGD, high-grade dysplasia; ESCC, esophageal squamous cell carcinoma.

Figure S1

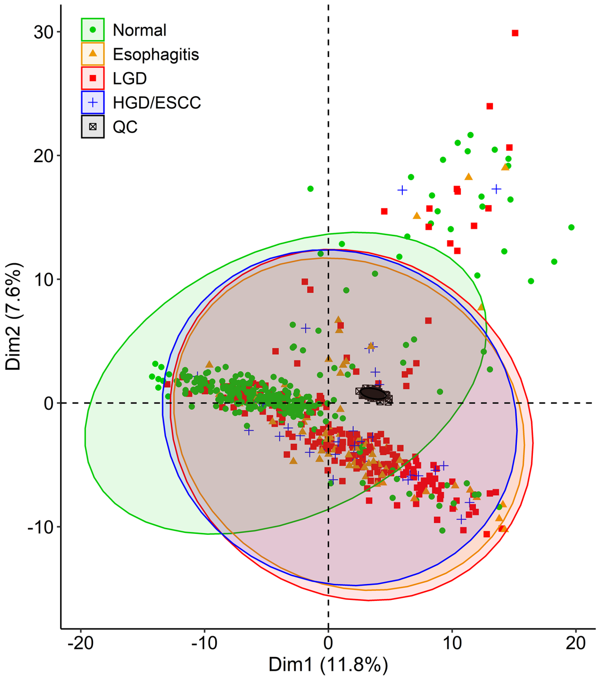
Normal
Esophagitis
LGD
HGD/ESCC
QC
30
20
10
0
-10
-20
-10
0
10
20
Dim2 (7.6%)
Dim1 (11.8%)

Figure S2

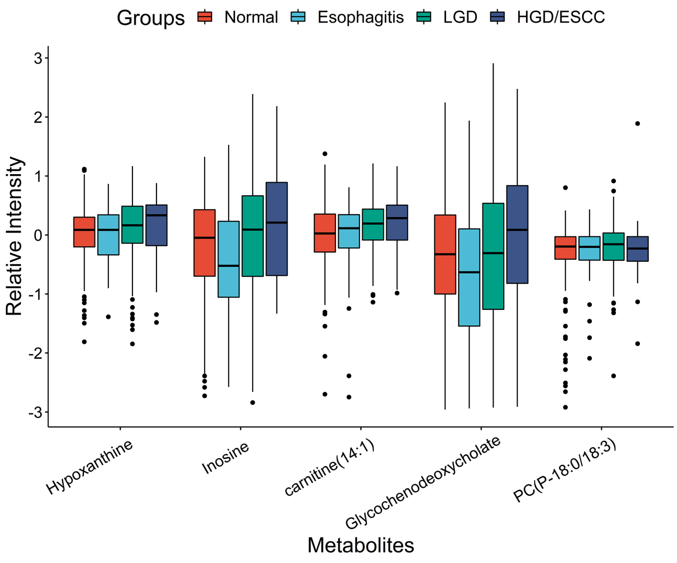

Groups
Normal
Esophagitis
LGD
HGD/ESCC
Relative Intensity
3
2
1
0
-1
-2
-3
Hypoxanthine
Inosine
carnitine(14:1)
Glycochenodeoxycholate
PC(P-18:0/18:3)
Metabolites

**Table S1** Metabolites associated with the progression of ESCC by univariate ordinal logistic regression analysis

| Metabolites | Cluster | p value | FDR | OR (95% CI) |
|---|---|---|---|---|
| Dopamine | 1 | 2.63E-05 | 1.17E-04 | 0.69 ( 0.59 ~ 0.82 ) |
| L-Histidine | 1 | 1.13E-09 | 2.26E-08 | 0.59 ( 0.49 ~ 0.70 ) |
| 5-Hydroxyindoleacetate | 1 | 8.73E-04 | 2.91E-03 | 0.78 ( 0.67 ~ 0.90 ) |
| L-Tryptophan | 1 | 1.50E-07 | 1.50E-06 | 0.67 ( 0.58 ~ 0.78 ) |
| 2'-O-methylcytidine | 1 | 1.99E-02 | 4.06E-02 | 0.70 ( 0.52 ~ 0.95 ) |
| PC (14:0/0:0) | 1 | 1.70E-06 | 1.30E-05 | 0.68 ( 0.58 ~ 0.79 ) |
| PC (O-16:1/0:0) | 1 | 6.42E-10 | 2.26E-08 | 0.61 ( 0.52 ~ 0.71 ) |
| PE (18:0/0:0) | 1 | 5.54E-08 | 7.38E-07 | 0.63 ( 0.53 ~ 0.74 ) |
| PC (16:1/0:0) | 1 | 1.95E-06 | 1.30E-05 | 0.68 ( 0.58 ~ 0.80 ) |
| PC (18:2/0:0) | 1 | 4.42E-04 | 1.61E-03 | 0.76 ( 0.65 ~ 0.89 ) |
| Hypoxanthine | 2 | 1.23E-03 | 3.77E-03 | 1.27 ( 1.10 ~ 1.47 ) |
| Inosine | 2 | 2.24E-05 | 1.12E-04 | 1.36 ( 1.18 ~ 1.57 ) |
| Carnitine (14:1) | 2 | 9.73E-05 | 3.89E-04 | 1.34 ( 1.16 ~ 1.56 ) |
| Glycochenodeoxycholate | 2 | 2.53E-03 | 7.22E-03 | 1.25 ( 1.08 ~ 1.44 ) |
| PC (P-18:0/18:3) | 2 | 1.88E-02 | 4.06E-02 | 1.19 ( 1.03 ~ 1.38 ) |

**Table S2** Pathway enrichment analysis for metabolites associated with the progression of ESCC

| Pathway Name | Total | Expected | Hits | Raw p | -log10(p) | Holm P | FDR | Impact |
|---|---|---|---|---|---|---|---|---|
| Tryptophan metabolism | 41 | 0.19 | 2 | 0.01 | 1.88 | 1 | 0.75 | 0.16 |
| Aminoacyl-tRNA biosynthesis | 48 | 0.22 | 2 | 0.02 | 1.75 | 1 | 0.75 | 0.00 |
| Purine metabolism | 65 | 0.29 | 2 | 0.03 | 1.50 | 1 | 0.89 | 0.02 |
| Histidine metabolism | 16 | 0.07 | 1 | 0.07 | 1.15 | 1 | 1.00 | 0.22 |
| beta-Alanine metabolism | 21 | 0.09 | 1 | 0.09 | 1.04 | 1 | 1.00 | 0.00 |
| Tyrosine metabolism | 42 | 0.19 | 1 | 0.18 | 0.76 | 1 | 1.00 | 0.13 |
| Primary bile acid biosynthesis | 46 | 0.21 | 1 | 0.19 | 0.72 | 1 | 1.00 | 0.01 |
